# Supplementary material for: Serine catabolism is essential to maintain mitochondrial respiration in mammalian cells
Source: Life Sci Alliance. 2018 May 21;1(2):e201800036. doi: 10.26508/lsa.201800036 (PMC6238390; doi:10.26508/lsa.201800036)
Supplement: Supplementary file 1 [file LSA-2018-00036_TableS1.pdf]

Table S1: sgRNA for editing SHMT

Species	Gene	Sequence
human	SHMT1	CATCTGCAATCTTCCGTAGC
human	SHMT1	TCAGGTGGCCCCCATCCGGA
human	SHMT2	CAACCTCACGACCGGATCAT
human	SHMT2	TCTCAGGATCACTGTCCGAC
mouse	SHMT2	GCTGGAGGTTGGACGGGCCAG
mouse	SHMT2	TCTGCAGAAGCTCCCACATCT
