# Supplementary material for: Serine catabolism is essential to maintain mitochondrial respiration in mammalian cells
Source: Life Sci Alliance. 2018 May 21;1(2):e201800036. doi: 10.26508/lsa.201800036 (PMC6238390; doi:10.26508/lsa.201800036)
Supplement: Supplementary file 2 [file LSA-2018-00036_TableS2.pdf]

Table S2: qPCR primer for human gene

Gene	Forward sequence	Reverse sequence
MT-ND1	ATGGCCAACCTCCTACTCCTCATT	TTATGGCGTCAGCGAAGGGTTGTA
MT-ND2	CTAGCCCCCATCTCAATCATA	GAATGCGGTAGTAGTTAGGAT
MT-ND3	CCCTACCATGAGCCCTACAAACAA	AGTCACTCATAGGCCAGACTTAGG
MT-ND4/4L	ACAAGCTCCATCTGCCTACGACAA	TTATGAGAATGACTGCGCCGGTGA
MT-ND5	ATCGGTTTCATCCTCGCCTTAGCA	ACCTAATTGGGCTGATTTGCCTGC
MT-ND6	AGGATTGGTGCTGTGGGTGAAAGA	ATAGGATCCTCCCGAATCAACCCT
MT-COI	ACCCTAGACCAAACCTACGCCAAA	TAGGCCGAGAAAGTGTTGTGGGAA
MT-CO2	ACAGATGCAATTCCCGGACGTCTA	GGCATGAAACTGTGGTTTGCTCCA
MT-CO3	ACTTCCACTCCATAACGCTCCTCA	TGGCCTTGGTATGTGCTTTCTCGT
MT-ATP6/8	TAGCCCACTTCTTACCACAAGGCA	TGAGTAGGTGGCCTGCAGTAATGT
MT-CYB	TCCTCCCGTGAGGCCAAATATCAT	AAAGAATCGTGTGAGGGTGGGACT
RNA28S	CCGTGCCTTGGAAAGCGTCGC	CAGAGGCTGTTCACCTTGGAGA
PPIA	GGCAAATGCTGGACCCAACACA	TGCTGGTCTTGCCATTCCTGGA
